# Acute Scleriasis; Spontaneous Recovery

**Published:** 1871-08

**Authors:** Henry G. Piffard

**Affiliations:** Surgeon to the New York Dispensary for Diseases of the Skin


*Acute Scleriasis; Spontaneous Recovery. Scleroderma; Treatment by Galvanization.* By Henry G. Piffard, M.D., Surgeon to the New York Dispensary for Diseases of the Skin.

Since Therial, in a monograph entitled "*Scleriase chez les Adultes*," first called attention to a singular affection of the skin, upwards of fifty cases, more or less closely resembling those described by him, have been recorded by different observers.

These cases may be readily divided into two distinct classes possessing but one or two prominent features in common; but, on the other hand, attended by circumstances which would seem to indicate that they have little, if any, pathological resemblance or connection; that, in fact, two distinct affections have been confounded under the same or similar names.

The most marked features to be observed in all of these cases are, a peculiar hardness and inelasticity of the integument, the firmness with which it is bound down to the subjacent tissues, and the impossibility of pinching it up into folds.

In the first class of these cases, we find that the invasion was rapid, that the lesion was extensive, usually occupying the greater portion of the integument of the upper half of the body and arms, the whole being involved in the course of a few days or weeks. Recovery generally took place in the course of a few months, under the influence of the most varied treatment, and even spontaneously. The microscope has failed to afford any clue to the nature of the affection.

In the cases of the second class, we notice that the affection is exceedingly chronic, and spreads from one or more points in patches or bands. It may occupy years in its progress, and is uninfluenced by the usual therapeutic agencies, many of those affected dying from intercurrent disease. The microscope has displayed well-marked changes in the texture of the tissues involved.

As full descriptions of these cases have been published and epitomized by different writers, we deem it hardly necessary to enter upon greater detail, but would refer to recent compilations by Dr. HILTON FAGGE, in *Guy's Hospital Reports for* 1867, and by Dr. W. de F. DAY, in the *Am. Journal of Med. Sciences*, July, 1869.

In the following cases we have confined the term *Acute Scleriasis* to an example of the first class, and the term *Scleroderma* to two examples of the second. These examples are reported for the sake of showing the contrast between the two diseases, and of drawing attention to a method of treatment which would seem to be appropriate to the latter affection.

### ACUTE SCLERIASIS.

David Gilbras, a native of Ireland, aged 49, and a blacksmith by occupation, came under the care of Dr. E. G. Janeway in the early part of the year 1869, suffering from emphysema of the lungs and chronic bronchitis. He remained under the care of Dr. J. for more than a year, without presenting any novel or peculiar features; but about the 1st of March, 1870, he noticed a stiffness on moving his neck. This was soon followed by a similar condition affecting the arms, back, chest, and abdominal region. The affection reached its height in about ten days, and then remained stationary for about four weeks longer, when it began to slowly decline. In April, Dr. J. kindly sent the case to the dispensary, and it came under my observation about six weeks after its first appearance. Upon examination, I found the integument of the parts above mentioned to be firmly adherent to the tissues beneath, but not discolored. It was impossible to lift or pinch it up in folds, or to cause it to glide over the underlying connective tissue. The patient was free from pain, and complained only of the inconvenience of being encased in an inelastic covering, which impeded the movements of his arms, and compelled the abandonment of his occupation. With the exception of his pulmonary difficulty, his organs and functions were in a normal condition. Careful inquiry failed to throw any light upon the determining cause of the attack, except, perhaps, frequent and sudden changes of temperature; for he stated that when overheated at the forge, he sometimes stood in a draught at the door to cool himself. Hoping and expecting to obtain some information by the aid of the microscope, I removed with a cutisector a thin section of skin from the side, an inch or two above the crest of the ilium. This spot was chosen, as it presented the most marked alteration, the affection having commenced to subside upon the upper portion of the chest and arms. The section was examined fresh in serum, and afterwards prepared and mounted in Damar. To my surprise, I was unable to detect any abnormal appearances.

The patient was placed upon small doses of quinine, in addition to the remedies prescribed by Dr. Janeway for his pulmonary difficulty, the quinine being given as a placebo, and to satisfy him that something was being done for his skin, rather than with a view to therapeutic result. The affection gradually subsided, and in three months recovery was complete.

### SCLERODERMA.

Of the following cases, the first is an abstract from a report by Dr. Fred Fieber, in the *Wiener Med. Wochenschrift*, Nov. 26th, 1870; the second came under my own observation at the Dispensary for Skin Diseases.

Dr. Fieber's patient was a girl of eleven years of age, and was admitted into the Vienna General Hospital on the 3d of May, 1869, with the following history:—Previous to the occurrence of the disease she had been frequently beaten, especially upon the left shoulder and the region of the chest. The child's father believed this to have been the cause of the disease. Eight months before her entrance into the hospital, there appeared over the left scapula a painless swelling or tumor, together with spots on the left upper extremity. The spots had brownish contours, and were accompanied with atrophy of the underlying muscles, a sclerema of the connective tissue corresponding to the left scapula, left breast, and whole of the left arm, which last seemed to be covered with a thick leathern sheath. The skin at some points was covered with scabs, and at others presented a cicatricial appearance.

Dr. Neumann made a microscopical examination, and found the appearances usually found in scleroderma.

Dr. Fieber believed the affection to be due to lesion of the trophic nerves, and commenced the treatment by galvanization of the sympathetic with 20 Daniel's elements, together with labile applications (30–40 El.) to the affected parts; in addition, Faradization of the atrophied muscles, with occasional steam baths. Electricity applied daily.

By the 30th of May, the skin had already become more supple.

By the first of June, the tissue along the external bicipital sulcus, to the breadth of half an inch, had become entirely soft, and, on the 5th of June, the sclerosis had lessened greatly in the scapular region. Progressive improvement occurred until the 10th of July, at which date the treatment was modified in the following manner: The left forearm was covered with diachylon, and the galvanic applications were confined to the upper arm, breast and shoulder. This was continued until the 14th of October, at the end of which time no further change had occurred in the tissues of the forearm, while the parts to which the current was steadily applied had become much softer.

On the 13th of February, 1870, there appeared near the right ear a patch of skin an inch long, and from two to three lines wide, which was deprived of pigment, thickened, and could scarcely be raised. This part received labile applications. By the 5th of March, the disease had almost entirely disappeared. On the 24th of March, without apparent cause, severe inflammation of the whole arm occurred, from which she recovered by the latter part of July.

Case II.—Johanna Achapt, born in Germany, aged 39 years, came to the Dispensary for Diseases of the Skin on the 20th of December, 1870, with the following history: Two years and a half before applying to the dispensary, she discovered that the skin behind the left malleolus was hard and unyielding, and felt like the calloused skin of a working man's hand. This condition continued, and began to extend up the outer side of the leg, in spite of various treatment. She occasionally suffered from pain in the affected limbs, but her chief complaint was on account of the impaired mobility and strength of the member. There was no appearance of the morbid condition upon any other region of the body. The general health was good.

On examination, I found that the skin behind the malleolus was hard, white, and its outermost layers disposed to loosen and curl up; these, however, could not be entirely removed without inflicting great pain. The skin was firmly bound down to the subjacent tissues, and tightly stretched as it were over the malleolus, and was apparently thinned and cicatricial in character. It could not be moved to the slightest degree over the bone.

Above the ankle the skin continued to present a sclerosed character, but appeared to be thicker than normal. As it was immovably attached, however, to the tissues beneath, its exact thickness could not be precisely determined. This condition extended up to the upper third of the leg, and occupied the greater portion of its external and posterior aspects. At the lower portion of the leg the peronei could not be distinguished from the neighboring border of the fibula. The surface of the skin was somewhat heightened in color, and presented a slight tendency to the exfoliation of epidermis. Sensation, tested by the æsthesiometer, indicated equal tactility as compared with corresponding points upon the other leg, but there was increased sensitiveness to pain, especially marked in those parts which were most profoundly altered. The temperature of the parts, as ascertained by a Seguin's surface thermometer, was 58° F. behind the malleolus; 74° at the middle; and 81° at the upper part of the leg. Corresponding points upon the healthy limb indicated 74½°, 78°, and 81° respectively. Above the knee the thermometer registered 89° on each limb. Four and a half inches above the ankle, the left leg measured 7½ inches in circumference, and the right 8 inches. This was apparently due to muscular atrophy from want of use.

Treatment was commenced upon the 20th of December by the application of the positive rheophore to the upper part of the left leg, and the negative was then moved slowly over the affected portions. It may be proper to state that at this time we were not aware of Fieber's case, or that galvanism had ever been applied in the affection we were treating. The applications lasted from five to ten minutes at each sitting, and were repeated every other day. Perceptible improvement was manifested after the fifth or sixth application of the continuous current. Improvement continued, and the patient was presented at the January and February meetings of the New York Dermatological Society. By the middle of March, the skin above the malleolus had resumed its normal aspect, and was freely movable upon the cellular tissue. The portion which lay immediately over the malleolus was also freely movable over the bone, and its cicatricial appearance was somewhat modified, having lost to some extent its dead white color. Considering the dermic condition as substantially well, we commenced Faradization of the gastrocnemius and other muscles. This was continued for a few days only, as the patient felt herself so much recovered that she did not deem it worth while to attend the dispensary any longer.

The battery employed consisted of a combination of thirty-two Smee's elements of small size, twenty-four of which gave *as intense a current* as the patient could bear without discomfort. It may be stated that galvanization of the sympathetic was tried on one or two occasions, but was not continued for fear of complicating the treatment. No medicines of any kind were given.

Clinical observation of the effects of electric currents in morbid conditions is so often at variance with the results of electro-physiological experiment, that very little assistance is afforded us in any endeavor to explain the former by a knowledge of the phenomena of the latter. We must therefore be excused from attempting to explain the *rationale* of the treatment employed in the cases recorded above. One fact, however, seems to be demonstrated, at least to the satisfaction of myself and of those who have witnessed the employment of electricity in cutaneous affections, and that is, that electrical currents, both primary and induced, possess in an eminent degree the power of modifying for the better certain morbid conditions of the integument. The problems yet to be determined are, what cutaneous affections are amenable to electrical treatment, and what *methods of application* are most likely to be followed by the desired results? It can hardly be expected that constant results will follow haphazard or purely empirical applications, and we conceive that the following, among other elements, must be taken into consideration, namely, the direction of the current employed, the position of the rheophores, the material of which they are composed, as affecting their conductibility, the length of application, and the dose as regards quantity and intensity. It is probable that the dose will be found to have an important influence in determining the result, and recent improvements in the mechanical construction of some of the batteries for both constant and induced currents, permit this to be measured with considerable accuracy. It is to be hoped that experimenters in this field, in announcing results, will record in full the procedures by which they have been obtained.—*Medical Gazette.*

